# aRNAque: an evolutionary algorithm for inverse pseudoknotted RNA folding inspired by Lévy flights

**DOI:** 10.1186/s12859-022-04866-w

**Published:** 2022-08-13

**Authors:** Nono S. C. Merleau, Matteo Smerlak

**Affiliations:** Max Planck Institute for Mathematics in the Sciences, Inselstrasse 22, 04103 Leipzig, Germany

**Keywords:** RNA inverse folding, Pseudoknotted RNAs, Evolutionary algorithm (EA), Lévy flight

## Abstract

**Background:**

We study in this work the inverse folding problem for RNA, which is the discovery of sequences that fold into given target secondary structures.

**Results:**

We implement a Lévy mutation scheme in an updated version of aRNAque an evolutionary inverse folding algorithm and apply it to the design of RNAs with and without pseudoknots. We find that the Lévy mutation scheme increases the diversity of designed RNA sequences and reduces the average number of evaluations of the evolutionary algorithm. Compared to antaRNA, aRNAque CPU time is higher but more successful in finding designed sequences that fold correctly into the target structures.

**Conclusion:**

We propose that a Lévy flight offers a better standard mutation scheme for optimizing RNA design. Our new version of aRNAque is available on GitHub as a python script and the benchmark results show improved performance on both Pseudobase++ and the Eterna100 datasets, compared to existing inverse folding tools.

**Supplementary Information:**

The online version contains supplementary material available at 10.1186/s12859-022-04866-w.

## Background

The function of non-coding RNA, which includes gene expression regulation (miRNAs, piRNAs, lncRNAs), RNA maturation (snRNAs, snoRNAs) and protein synthesis (rRNAs, tRNAs), strongly depends on the hierarchical folding of RNA molecules. Given their sequence of bases (primary structure), RNAs fold into secondary structures, such as stem loops and pseudoknots, before folding into higher level (tertiary and quaternary) structures. The secondary structure is considered to be a list of base pairs. The base pairs can include canonical (Watson-Crick pairs [1, 2]), non-canonical interactions, and crossing or pseudoknot interactions [3]. Compared to other interactions, non-canonical interactions occur with reduced frequency. The crossing or pseudoknot interactions occur when two canonical or non-canonical interactions cross each other [3].

Considering pseudoknots in designing functional RNAs is vital given their role in realising biological functions. In modern bio-engineering, one must solve the RNA inverse folding problem to be able to design RNA molecules performing specific functions [4–6]. Here, we consider the RNA secondary structure inverse folding problem. The goal is to find RNA sequences that fold into a given target secondary structure, with or without pseudoknots.

A key prerequisite to addressing the RNA inverse problem is a reliable solution to the folding problem. Computationally folding an RNA molecule consists of searching in the space of all possible secondary structures for one that minimises the free energy. Designing sequences for a pseudoknotted target structure is computationally more expensive than a pseudoknot-free target because of the folding algorithms’ complexity. Specifically, the time complexity of the pseudoknot-free secondary structure prediction is $O(n^3)$ when using dynamic programming approaches such as RNAfold [7, 8], or less with heuristic folding methods (e.g. *O*(*n*) for LinearFold and $O(n^2\log n)$ for RAFFT [9]). By contrast, when considering a special class of pseudoknots, the time complexity of folding goes up to $O(n^6)$ for an exact thermodynamic prediction using a dynamic programming approach such as [10]. In this work, we consider only two heuristics tools (IPknot [11] and HotKnots [12]) chosen for their lower time complexity $O(n^4)$.

Many of the studies addressing the inverse folding of RNA considered only pseudoknot-free secondary structures [13–26]. There are, however, four exceptions: MCTS-RNA [24], antaRNA [27], Modena and Inv [28]. MCTS-RNA uses a Monte Carlo tree search (MCTS) technique. This technique has recently shown exceptional performance in Computer Go. It is used to initialise the RNA sequences solutions in MCTS-RNA and the solutions are further improved through local updates at the nucleotide positions. MCTS-RNA uses pkiss as a folding tool, whereas the older tools support a broader range of folding tools. antaRNA utilises an ”ant-colony” optimisation technique. The technique begins with an initial population of sequences generated via a weighted random search; next, the solutions are evaluated, and the sequence fitness values are used to refine the weights and improve the sequences over generations. Another approach (Modena) implements a multi-objective evolutionary algorithm measuring both the stability of the designed sequences and the similarity of folded sequences to the target structure. Although the first version of Modena was implemented for pseudoknot-free structure [17], it has since been extended to support pseudoknotted RNAs, and a new crossover operator [29]. Inv is the first inverse folding tool handling pseudoknotted RNA target structures but is restricted to a specific type of pseudoknot pattern called 3-crossing nonplanar pseudoknots.

Since the genetic algorithm (or more generally evolutionary algorithm) was proposed by John Holland [30] in the early 1970s, it has emerged as a popular search heuristic and found application in many disciplines that deal with complex landscape optimisation problems. In a prior publication [31], we presented aRNAque, a simple evolutionary inverse folding algorithm guided by local (or one-point mutations). Although a local search can efficiently discover optima in a simple landscape, more complex landscapes pose challenges to the design of evolutionary algorithms that rely solely on local search. This is especially true on a landscape with high neutrality where local search may be inefficient or risk getting stuck on a plateau (or local optimum). To avoid this pitfall, we propose here an extension of aRNAque which implements a new mutation scheme inspired by Lévy flights (called Lévy mutation) and supports pseudoknotted RNA target structures.

Lévy flights are random walks with a Lévy (or any heavy-tailed) step size distribution. The concept originates in the work of Mandelbrot on the fluctuation of commodities prices in the 1960s [32] but has since found many more physical applications [33]. The term ”Lévy flight” was also coined by Mandelbrot, who used one specific distribution of step sizes (the Lévy distribution, named after the French mathematician Paul Lévy). Lévy flights also play a key role in animal foraging, perhaps because they provide an optimal balance between exploration and exploitation [34, 35]. For a recent review of applications of Lévy flights in biology from the molecular to the ecological scale, [36].

Similar to a Lévy flight, a Lévy mutation scheme allows simultaneous search at all scales over the landscape. New mutations often produce nearby sequences (one-point mutations) but occasionally generate mutant sequences far away in genotype space (macro-mutations). In this work, the number of point mutations distribution at every step is taken to follow a Zipf distribution [37].

The optimisation approach implemented in aRNAque is an evolutionary algorithm, which consists of a population of RNA sequences that all perform separate random walks (are mutated) in the space of possible sequences, and whose step sizes (number of point mutations) follow a Zipf distribution. After each step, the probability of surviving is proportional to the fitness of each sequence, which is evaluated by its ability to approximate a given target structure. We provide a brief overview of that approach in the following subsection.

Earlier works have applied similar ideas in genetic programming [38], and in differential evolutionary algorithms [39]. This has motivated us to investigate the possible benefit of a Lévy flight in designing RNA sequences. Using a Lévy mutation scheme, we aim to speed up our prior evolutionary algorithm and increase the diversity of the designed RNA sequences.

We compared the performance of our modified version of aRNAque to existing tools through a benchmark on two well-known RNA datasets: PseudoBase++ [40] for the pseudoknotted targets and Eterna100 [41] for the pseudoknot-free targets. On the PseudoBase++ dataset, the difference between the local mutation and the Lévy mutation concerning the number of generations (or evaluations) was significant (with a *p* value $\approx 0.00004$). Using the two pseudoknot folding tools HotKnots and IPknot, our designed sequences were of better quality than the ones produced by antaRNA regarding the average base pair distance to the desired targets. We performed a second benchmark on the Eterna100 dataset. Considering the Eterna100-V1 dataset, the Lévy mutation scheme solved 89 targets out of 100 whereas the local mutation scheme solved 91/100. Combining the two benchmark results obtained using both mutation schemes, we counted the number of distinct targets solved by aRNAque. aRNAque designs successfully 92/100 of the Eterna100-V1 dataset and 94/100 of the Eterna100-V2 dataset.

## Implementation of **aRNAque**

aRNAque implements an evolutionary algorithm approach. Below, we provide a brief overview of our evolutionary search algorithm and our mutation scheme.

### Overview

In general, an evolutionary search algorithm on any fitness landscape consists of three main parts, which in the context of RNA inverse folding are as follows:

- Initialization: generating a random initial population of RNA sequences compatible with the given target secondary structure.
- Evaluation and selection: evaluating a population of RNA sequences consists of two steps: (1) fold each sequence into a secondary structure and assign it a weight based on its similarity to the target structure. (2) select a weighted random sample with replacement from the current population to generate a new population. A detailed description of the objective function used in aRNAque is provided in [31].
- Mutation (or move) operation: define a set of rules or steps used to produce new sequences from the selected or initial ones. This component is elaborated further in the following subsection.

aRNAque’s pseudocode is provided in the Additional file 1 (see SI 4).

### Mutation mode

For a given target RNA secondary structure $\sigma ^*$ of length *L*, the space of potential solutions to the inverse folding problem is $S=\{\text {A},\text {C},\text {G},\text {U}\}^L$. An evolutionary algorithm explores the space *S* through its move (or mutation) operator.

Given a sequence $\phi \in S$, a sequence $\phi ' \in S$ is said to be an *n*-point mutation of $\phi$ if it differs from $\phi$ at *n* nucleotides; i.e. $h(\phi , \phi ')=n$ where *h*(., .) is the hamming distance on *S*.

A mutation mode is a random variable *U* taking values in $\{1,...,L\}$. We define *P*(*U* = *n*) as the probability that exactly *n* nucleotides, selected uniformly at random, undergo point mutation during a mutation event. *U* can generally be any probability distribution. We examined the binomial and Zipf distributions for local and Levy search, respectively:

- Binomial mutation: *U* has a binomial distribution:
  $$\begin{aligned} P(U=n)= \left( {\begin{array}{c}L\\ n\end{array}}\right) \mu ^n (1-\mu )^{L-n} \end{aligned}$$
  for some $0 \le \mu \le 1$, such that the average number of point mutations $u=\mu \cdot L$. We can think of this mutation mode arising from each nucleotide of an RNA sequence independently undergoing a point mutation with probability $\mu$, i.e. $\mu$ is the per-nucleotide or point mutation rate.
- Lévy mutation: *U* has a Zipf distribution given by:
  $$\begin{aligned} P(U=n)= \frac{1/n^c}{ \sum _{k=1}^{L}{1/k^c}} \end{aligned}$$
  Where $c>0$ is the value of the exponent characterizing the distribution. Larger values of *c* are associated with a more significant proportion of local search, while smaller values of *c* imply a more considerable proportion of long-range search.

**Fig. 1:**
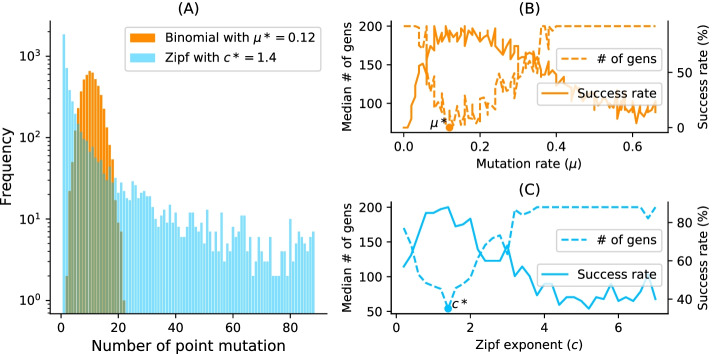
Binomial vs. Zipf distributions. **a** Samplings Binomial and Zipf distributions for the best binomial mutation rate $\mu ^*$ (respectively $c^*$ for the best Zipf exponent parameter). Both distributions have a mean of 8.7 point mutations for a sequence of length 88 nucleotides. **b** Tuning of binomial mutation rate parameter. For each $\mu \in [0,1]$ with a step size of 0.005 and the pseudoknotted target PKB00342 of length 88, 50 sequences were designed using aRNAque. (**b**) shows the median generations and the success percentage vs. the mutation rate ($\mu$). The best mutation rate is $\mu ^*=0.085$ (with a median number of generation 93.5 and a success rate of 92%). **c** Tuning of Levy exponent. Similar to (**b**), for each $c \in [0,7]$ with a step size of 0.1 and for the same pseudoknotted target structure, 100 sequences were designed using aRNAque. It shows the median generations and the percentage of success vs. the exponent parameter (*c*). The Zipf exponent distribution that produced the highest success rate and the minimum number of generations is $c^*=1.4$

Figure 1 shows the distribution of the number of point mutations on a sequence of length 88 nucleotides for both mutation schemes. Both distributions have the same mean but differ markedly in their tails.

Throughout this paper, the local mutation will refer to binomially distributed mutation with parameter $\mu \approx 1/L$ or one-point mutation.

### New feature

We provide an updated version of aRNAque supporting pseudoknotted RNA target structures. In addition to the support for pseudoknots, we provide an updated mutation mode based on a Zipf distribution. We present the mutation algorithm in Algorithm 1.

**Figure:**
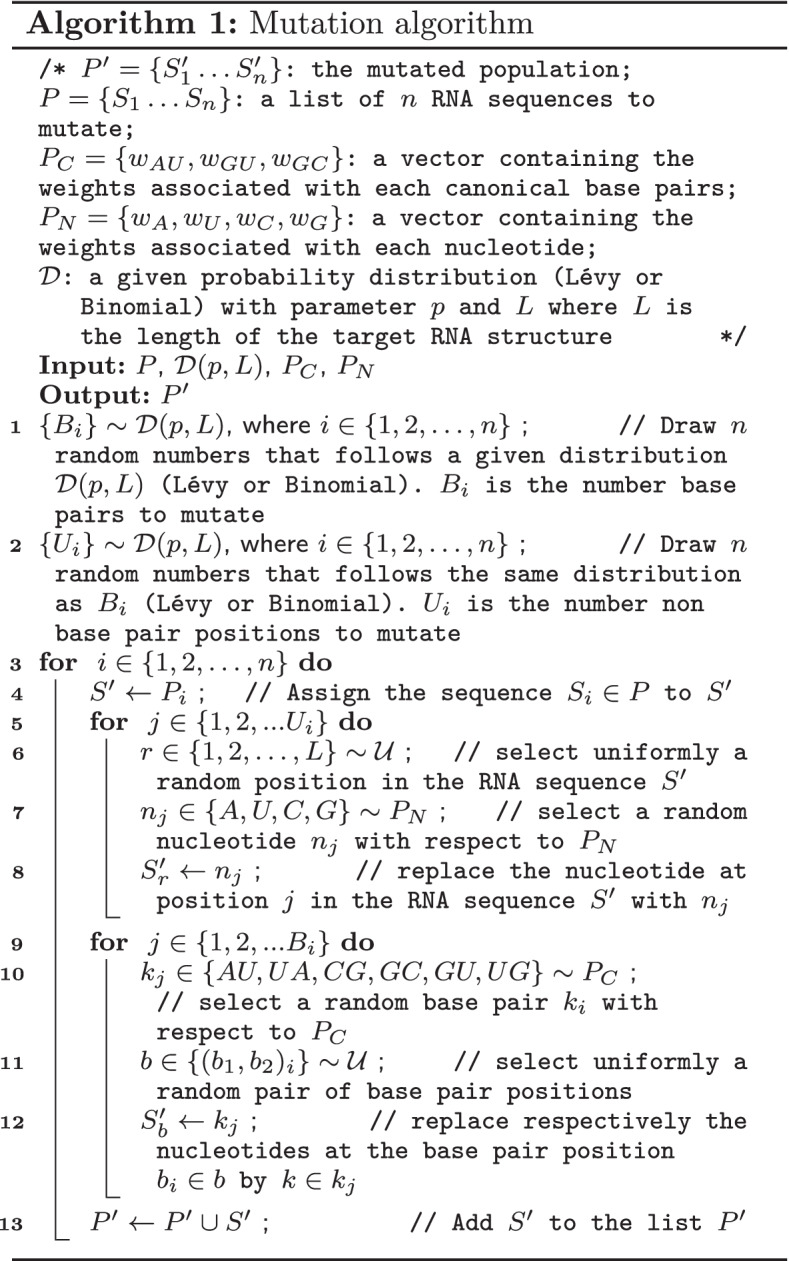

## Parameter analysis and benchmark data

Here we analyse mutation parameters and compare local and Lévy mutation modes.

### Benchmark data

To compare our new version of aRNAque with existing tools in the literature, we used the PseudoBase++ benchmark datasets for pseudoknotted target structures and the Eterna100 dataset for pseudoknot-free target structures.

**Fig. 2:**
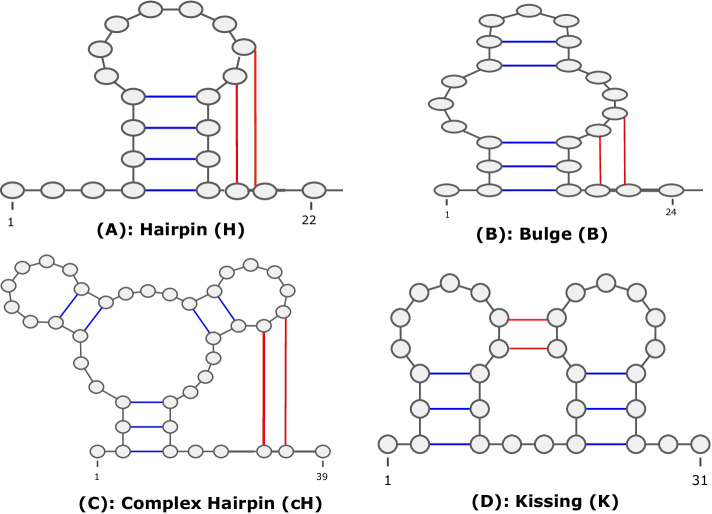
Types of pseudoknots accommodated by aRNAque. **a** Hairpin (H-type) pseudoknot. **b** Bulge (B-type) pseudoknot. **c** Complex hairpin (cH-type) pseudoknot. **d** Kissing hairpin (K-type) pseudoknot. The B-type and cH-type are all complex form of H-type. The pseudoknot interactions are highlighted in red

The PseudoBase++ is a set of 266 pseudoknotted RNA structures used to benchmark Modena. It was initially 342 RNA secondary structures, but the redundancy and the non-canonical base pairs 76 structures were excluded. To group the dataset with respect to the pseudoknot motifs (Fig. 2), we used the test data from antaRNA’s paper. The test data contains 249 grouped into four categories: 209 hairpin pseudoknots (H), 29 bulge pseudoknots (B), 8 complex hairpin pseudoknots (cH) and 3 kissing hairpin pseudoknots (K). Out of the 266 structures, only 185 (with 150 H-type, 3 K-type, 25 B-type and 7 cH-type) structures were included in the test data. So for that reason, we have used only 185 target structures for the pseudoknot motif performance comparison and the 266 structures for the different target lengths performance comparison.

The $\texttt {Eterna100}$ dataset [42] is available in two versions and both contain a set of $100$ target structures extracted from the EteRNA puzzle game and classified by their degree of difficulty. The Eterna100-V1 was initially designed using ViennaRNA 1.8.5, which relies on Turner1999 energy parameters [43]. Out of the 100 target secondary structures, 19 turned out to be unsolvable using the version of ViennaRNA 2.4.14 (which relays on the Turner2004 [44]). Subsequently, an Eterna100-V2 [42] was released in which the 19 targets were slightly modified to be solvable using ViennaRNA 2.4.14 and any version that supports the Turner2004 energy parameters. The main difference between the two dataset relay on the energy parameters used to generate the data.

### Methodology

#### Folding tools

This work considers two pseudoknotted RNA folding tools: HotKnots and IPknot. For pseudoknot-free RNA folding, we used RNAfold. For the mutation parameter and GC-content analysis presented in our work, we used IPknot, and both HotKnots and IPknot for PseudobBse++ benchmarks. To be able to use HotKnots in aRNAque without copying aRNAque in the bin directory of Hotknots, we have performed some modifications on Hotknots source code. Details on the modifications are provided in the Additional file 1: SI 6. Furthermore, we considered pkiss, a well known tool for K-type pseudoknot prediction, but since the PseudoBase++ dataset contains just 4 K-type pseudoknotted structures and pKiss has higher time complexity ($O(n^6)$), we did not find it efficient for the benchmark we performed.

#### Mutation parameters tuning

An evolutionary algorithm’s main challenge is to find optimum parameters such as mutation rate, population size and selection function. We used 80 pseudoknotted targets with lengths from 25 to 181 nucleotides for the mutation parameter analysis. We set the maximum number of generations *T* to 200 and the population size *n* to 100. The stopping criteria are two: 1) the number of generations (*t*) is equal to the max number of generations (*T*) or 2)the minimum hamming (or base pair) distance of the best RNA sequence solution to the target is 0. The best mutation parameters ($c^*$ for Levy and $\mu ^*$ for Binomial) are those that have the lowest median number of generations. The best mutation parameters obtained for both binomial and Lévy mutation modes are used to benchmark and compare the results on the entire datasets of RNA structures.

#### Benchmark on the **PseudoBase++** dataset

Four benchmarks are performed on the pseudoknotted dataset: 1) mutation parameter analysis, 2) the GC-content and diversity analysis, 3) Local search versus Lévy search, 4) aRNAque (Lévy search) versus antaRNA. For the aRNAque (Binomial and Lévy) case, the four benchmarks share the same number maximum number of generations (*T* = 200), population size (*n* = 100), stopping criteria (*t* = *T* or min fitness equals 0). We used a T-test to compare local-search (Binomial with low mutation rate) means of the number of generations distributions to the ones of the Lévy search. The *p* values and *t*-values are presented in the result section. For the antaRNA benchmark, the maximum number of iterations was set to 1200, and a slight modification was made to allow the support of the folding tool $\texttt {HotKnots}$ (See Additional file 1: SI 6). For booth tools and each benchmark, 20 runs were launched independently in parallel on a computer with the same resources, resulting in 20 designed sequences per pseudoknotted target structure. Each designed sequence s is folded into a secondary structure $\sigma$, and the similarities between $\sigma$ and $\sigma ^*$ are computed using the base pair distance. For the GC-content benchmark, four GC-content values are considered, $\{0.25, 0.5, 0.75,1\}$ and the setting of each tool reminds the same.

#### Benchmark on the **Eterna100** dataset

We performed two benchmarks are one the Eterna100 dataset: 1) a benchmark on the Eterna100-V1 dataset using the Turner1999 energy parameter and the both versions of aRNAque (one point and Lévy mutation), 2)a benchmark on the Eterna100-V2 dataset using the Turner2004 energy parameter and both versions of aRNAque (one point and Lévy mutation). For each of the Eterna100 benchmark we used the same evolutionary algorithm parameters; a maximum of *T* = 5000 generations (i.e. a maximum of 500,000 evaluations), a population size of *n* = 100 and the same stopping criteria (the number of generation *t* = *T* or min fitness equals 0). For local and Lévy search, 5 runs were launched independently, resulting in 5 designed sequences per target. We define success rate simply as the number of successfully designed targets. A target is considered successfully designed when at least one of the designed sequences folds into the target structure.

## Results

We first compared the performance of aRNAque using Lévy mutations to the previous version with local mutations. Secondly, we compared aRNAque to the existing pseudoknotted RNA inverse folding tool antaRNA using two folding tools: HotKnots and IPknot. Finally, we compared the performance of aRNAque (Lévy mutation) to the one of Ivry et al. on a tripod RNA secondary structure.

### Analysing the best mutation parameter on **PseudoBase++**: Levy mutation vs. local mutation

The advantage of using a Lévy mutation is its capacity to allow a simultaneous search at all scales over the landscape. The search at different scales is often dictated by the exponent parameter of the heavy-tailed distribution. In this first result section, we analyse the distributions of the best mutation parameters for 80 pseudoknotted target structures and for both mutation schemes.

**Fig. 3:**
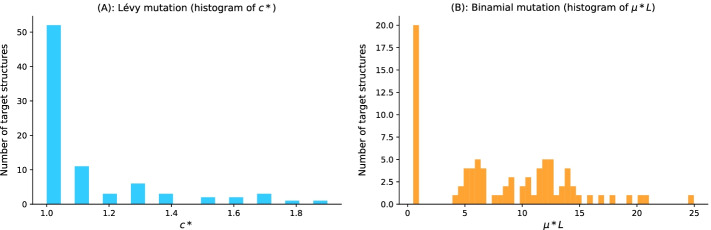
Parameter tuning for both binomial and Lévy mutation schemes. **a** Lévy mutation parameter tuning. Histogram of best exponent parameter ($c^*$) for a set of 81 target structures with different pseudoknot patterns and various lengths. The most frequent best exponent value is 1. **b** Binomial parameter tuning. Histogram of best mutation rate ($\mu ^*$) for the same set of 81 target structures with different pseudoknots and various lengths. The most frequent best parameter is the low mutation rate ($\approx 1/L$). For some structures, the best mutation rate is the high one for different lengths as well

- Binomial mutation: From Fig. 1b, the critical range was identified to be from 0 to 0.2, and as $\mu$ becomes greater than 0.1, the success rate decreases and the average number of generations increases. For each of the 80 target structures with pseudoknots, 20 sequences were designed for $\mu \in [0,0.2]$ with a step size of 1/*L*. Figure 3b shows the histogram of the best mutation rate found for each target structure. Two main regimes are apparent: one where the best mutation rate is a shallow mutation rate ($\approx 1/L$) and another where the high mutation rate is optimal.
- Lévy mutation: From Fig. 1c, the critical range of *c* was identified to be [1, 2]. For $c \in [1,2]$ and a step size of 0.1, an optimum exponent parameter $c^*$ was investigated for all the 80 target structures. Figure 3a shows the histogram of $c^*$. Contrary to binomial mutation, the optimum exponent parameter does not vary too much ($\forall \sigma$, $c^*\approx 1$).

Figure 3 shows that when using a Lévy mutation, the optimal mutation rate is the same for most target structures. In contrast, the optimum binomial mutation rate parameter $\mu ^*$ mostly varies with different targets. Although both mutation schemes (for the best mutation parameters) have approximately the same success rates, the Lévy flight mutation scheme is more robust to different targets.

### Performance on **PseudoBase++**: Levy mutation vs. local mutation

**Fig. 4:**
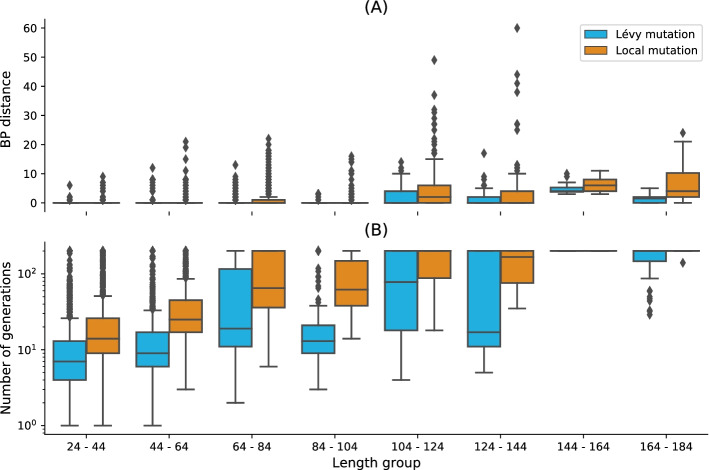
Lévy mutation mode vs local mutation (one-point mutation). **a** Hamming distance distributions vs. target structure lengths. **b** Number of generations distributions for different length groups. In both (**a**) and (**b**), lower values indicate better performance. The target structures are solvable in less than 100 generations for both mutation schemes and most length groups. Still, the difference in the number of generations gets more significant as the target lengths increase, except for the two last length groups for which both mutation schemes mostly failed. The highest difference in terms of median number of generations is 150 for target lengths in the range [124–144] (respectively 123, 49, 46, 16, 7, 0, 0 for the length ranges [84–104], [64–84], [104–124], [44–64], [24–44], [144–164], [164–184]). Averaging over all length groups, the median number of generations difference between the Levy mutation and the one point mutation is 48 generations

Figure 4 shows box plots for the base pair distance (Hamming distance) and the number of generations for increasing target lengths under our two mutation schemes: binomial at low mutation rate (or one point mutation) and the Lévy mutation. For each pseudoknotted RNA target structure in the PseudoBase++ dataset, we designed 20 sequences. The results show that using the Lévy mutation instead of a local mutation scheme can significantly increase the performance of aRNAque. The gain was less significant in terms of designed sequences quality (base pair distance distributions, with a *t* value $\approx -1.04$ and *p* value $\approx 0.16$) but more significant in terms of the average number of generations needed for successful matches to target structures (with a *t* value $\approx -3.6$ and *p* value $\approx 0.0004$). This result demonstrates a substantial gain in computational time when using a Lévy mutation scheme instead of a purely local mutation.

### Performance on **PseudoBase++**: **aRNAque** vs. **antaRNA**

#### General performance analysis using both **Hotknots** and **IPknot**

**Fig. 5:**
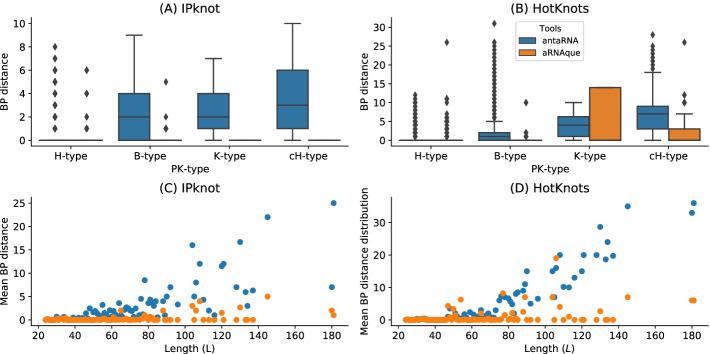
aRNAque vs antaRNA on PseudoBase++ dataset using both IPknot and HotKnots. Lower values imply better performance. **a, b** Base pair distance distributions of the designed sequences to the target structure for different pseudoknot types. **c, d** Mean base pair distance against target lengths

We also compared the sequences designed using aRNAque (with the Lévy mutation scheme) to those produced by antaRNA. Figure 5a, c show the base pair distance distribution for each category of the pseudoknotted target structure and the mean of the base pair distance plotted against the length of the target secondary structures. For antaRNA, and when using IPknot as a folding tool, finding sequences that fold into the target becomes increasingly tricky with pseudoknot complexity (median base-pair distance distribution increases). On the other hand, aRNAque’s performance improves as pseudoknot complexity increases (e.g. the mean base-distance decreases with the pseudoknot complexity).

We performed a second benchmark on the same dataset using HotKnots as a folding tool. For both aRNAque and antaRNA, the more complex the pseudoknot motifs, the worse is the tool performance (median of the base-pair distance distribution increases). Figure 5b, d show respectively the base pair distance distributions with respect to the pseudoknot motifs, and the mean of the base pair distance respect to the length of the target structures for both aRNAque and antaRNA. Even though both performances degrade as target length increases, aRNAque (Lévy flight evolutionary search) performance remains almost constant for all the target lengths greater than 60.

#### GC–content analysis of the designed sequences using **IPknot**

**Fig. 6:**
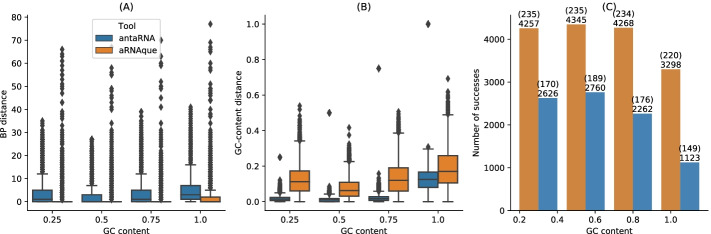
aRNAque vs antaRNA on PseudoBase++ dataset using IPknot: GC–content analysis. **a** Base-pair distance ditributions. **b** GC–content distance distributions. The difference betwen the targeted GC-content and the actual GC-content values. In (**a**, **b**), lower values imply better performance. **c** Number of successes realised by both inverse folding tools. Two values are considered: the up value represent the number targets successfully solved for each GC-content value out of the 266 targets benchmarked; the down values represent the number sequences folding into the targeted secondary structure

The GC–content of an RNA sequence *S* measures the concentration of G-C nucleotide in *S* and influences its stability and biological function [45–47]. Therefore, the ability of an inverse folding tool to control the GC–content is of vital importance for designing functional RNA sequences. Both antaRNA and aRNAque allow to control the GC–content at different levels of the optimization process: aRNAque through the mutation parameters $P_C$ and $P_N$; antaRNA with the parameter $tGC\in [ 0,1 ]$. In this section, we compare the performance of each tool for fixed GC–content values and analyse each tool’s ability to control the GC–content. For each pseudoknotted target structure in the PseudoBase++ dataset, four different GC-content values $\{0.25,0.5, 0.75, 1\}$, a pool of 20 sequences is designed using IPknot as folding tool. That results in 5320 designed sequences for each GC–content value and tool. The number of successes is the total number of sequences that fold exactly into the given target structure (i.e. the designed sequence folds into a structure at base-pair distance 0 from the target structure). Figure 6 shows respectively the base pair distance distributions, the GC distance distributions and the number of successes for both aRNAque and antaRNA. The results show that the performance (in terms of success number) varies considerably with the GC–content values for both tools, and the best performance is obtained for both tools with a GC–content value of 0.5. When comparing the GC-content distance (i.e absolute value of the difference between the targeted GC–content and the actual GC–content values of the designed sequences) distributions, both GC–content distance median distributions increase, whereas antaRNA controls significantly better the GC-content (See Fig. 6b). On average, for the respective GC-content values $\{0.25, 0.5, 0.75, 1\}$, antaRNA’s sequences have respectively 0.2569, 0.4952, 0.7314, 0.8684 whereas aRNAque’s sequences have respectively 0.3649, 0.4910, 0.6231, 0.811; the main difference is at fixed GC-content values 0.25 and 0.75. Even though antaRNA designs sequences with better control of the GC-content, the gap in success rate still remains remarkable compared to aRNAque(See Fig. 6a, c).

#### Diversity of the designed sequences

**Fig. 7:**
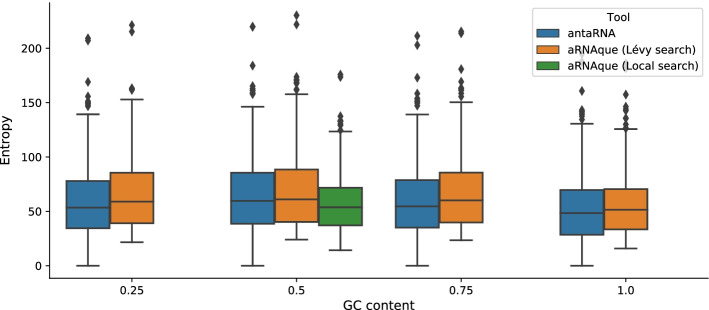
aRNAque vs antaRNA on PseudoBase++ dataset using IPknot: Diversity analysis. The positional entropy distributions plotted agains the targeted GC–content values. Higher values imply better performance

Another advantage of using a Levy mutation when designing RNA sequences is to increase the chance of designing sequences with high diversity. Here, we use the positional entropy of each pool of 20 sequences previously designed for each pseudoknotted target structure to compare the diversity of RNA of both tools antaRNA and aRNAque (Lévy search). We also compare it to the diversity of the designed sequences using the old version of aRNAque (Local search). The results show that the sequence diversity of both antaRNA and aRNAque (Lévy search) varies with the GC–content values, where the more diversified pool of sequences is achieved with a GC–content value of 0.5. When comparing the pool of designed sequences with highest entropy (i.e. with a fixed GC-content of 0.5) to the one of the old version of aRNAque (Local search), the aRNAque (Lévy search) and antaRNA produce sequences with similar entropy (i.e. with a median entropy of 61.01 for Lévy search respectively 59.65 for antaRNA (see Fig. 7), whereas the entropy of the sequences designed using the Local search is lower. For the three others fixed GC-content values (i.e. $\{0.25, 0.75, 1 \}$, aRNAque (Lévy search) produces sequences with the highest entropy (respectively a median entropy of 58.9, 60.08, 51.52 against 53.42, 54.63, 48.38 for antaRNA).

#### CPU time versus success rate analysis using **Hotknots**

**Fig. 8:**
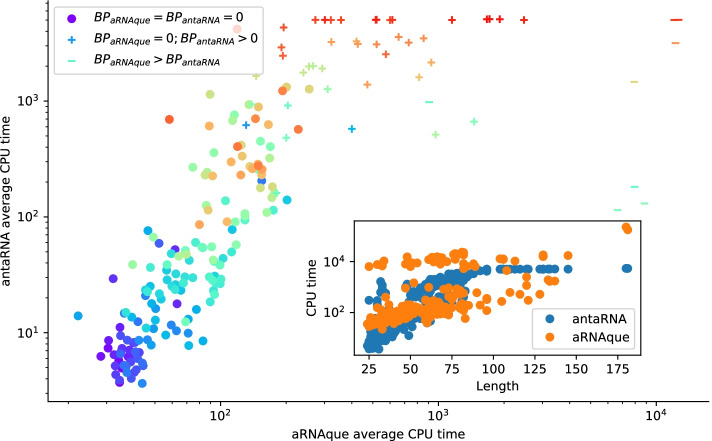
CPU time analysis using Hotknots: antaRNA vs. aRNAque. Each bubble corresponds to a target structure in PseudoBase++ dataset and, their colours are proportional to the length of the targets. In the legend, BP stands for Median base pair distance, and the different markers represent—(’o’) 100% success for both tools—(’+’) 100% success for aRNAque and not for antaRNA—(’–’) for the case aRNAque’s desinged sequences are of median base pair distances greater than the one of antaRNA. Underlying the CPU time difference is the inside plot that shows the CPU time (in s) with respect to the target length

We also compare aRNAque’s computational time to the one of antaRNA. For both tools, 20 sequences were designed for each target structure of the PseudoBase++ dataset. The GC–content value used for both tools is 0.5, and the maximum number of interactions for antaRNA is 5000. Figure 8 shows the median CPU time of the 20 runs in seconds for both tools plotted against each other. We analysed the CPU time by partitioning the data into three groups: 1) a set for which both tools have a median base-pair distance of 0 (158 entries marked with o); 2) another set for which aRNAque has a median base-pair distance of 0 and antaRNA (41 entries marked with +); 3) the last set for which antaRNA designs are of better quality (9 entries mark as -). For the first group, we can notice that for most targets of short length antaRNA is faster than aRNAque. For the second group, although antaRNA average CPU time remains smaller, aRNAque’s success rate outperformed antaRNA. On the one hand, aRNAque average CPU time is higher than the one of antaRNA, but this could be due to its population-based algorithm, which often allows for designing more successful sequences. On the other hand, antaRNA is faster but less successful. Increasing antaRNA’s number of iterations will indeed increase the CPU time, but it may improve the quality of the designed sequences.

### Performance on **Eterna100** dataset

We performed a third benchmark on the Eterna100 datasets. First, on the Eterna100-V1 dataset, the Lévy flight version of aRNAque successfully designed 89% of the targets, and the one-point mutation (local mutation) version achieved 91% of success, suggesting that for some target structures, local mutation can outperform the Lévy mutation scheme. Combining the two solutions, aRNAque solved in total 92% of the targets of Eterna100-V1 (see also [31]).

**Fig. 9:**
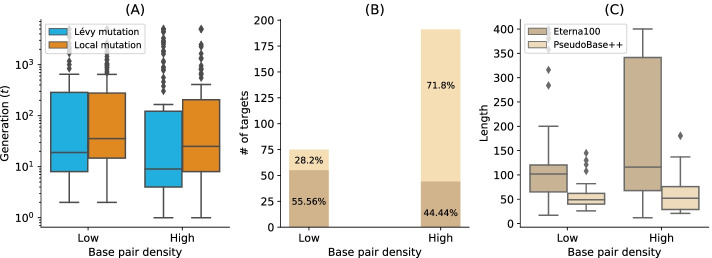
Lévy mutation vs. Local mutation: performance analysis with respect to the base-pair density. The higher the base-pair density is, the more useful the Lévy mutation scheme to speed up the optimization EA. **a** Distributions of number of generations for the low and high base-pair density targets of the Eterna100 dataset. **b** Percentages of targets with low and high base-pair density for the Eterna100 and PseudBase++. **c** The length distributions of the low and high base-pair density pseudoknot-free and pseudoknotted targets

When analysing the performance of Lévy flight for low and high base pair densities separately, the median number of generations of high base pair density targets was lower than the one with low base-pair density (8 generations for high density and 18 for the low base pairs density targets). The same observation was drawn for the success rate. For the low base-pair density targets, the Lévy flight achieved 87% (49/56) success, whereas, for the high base-pair density, it achieved 91% (40/44). The same analysis can be done when comparing the one-point mutation results for the high-density targets to the Lévy flight mutation. The median number of generations for the low-density targets when using a one-point mutation operator was 34 (respectively 24 for the high base pair density targets) (see Fig. 9a).

A new benchmark was performed on Eterna100-V2 with aRNAque achieving a 93% success rate when combining the designed solutions for both mutation schemes. Compared to recently reported benchmark results [42], aRNAque achieved similar performance to NEMO on Eterna-V2: one target was unsolved by all existing tools and one target solved only by NEMO remained unsolved by aRNAque.

### **aRNAque** performance on a tripod secondary structure

**Fig. 10:**
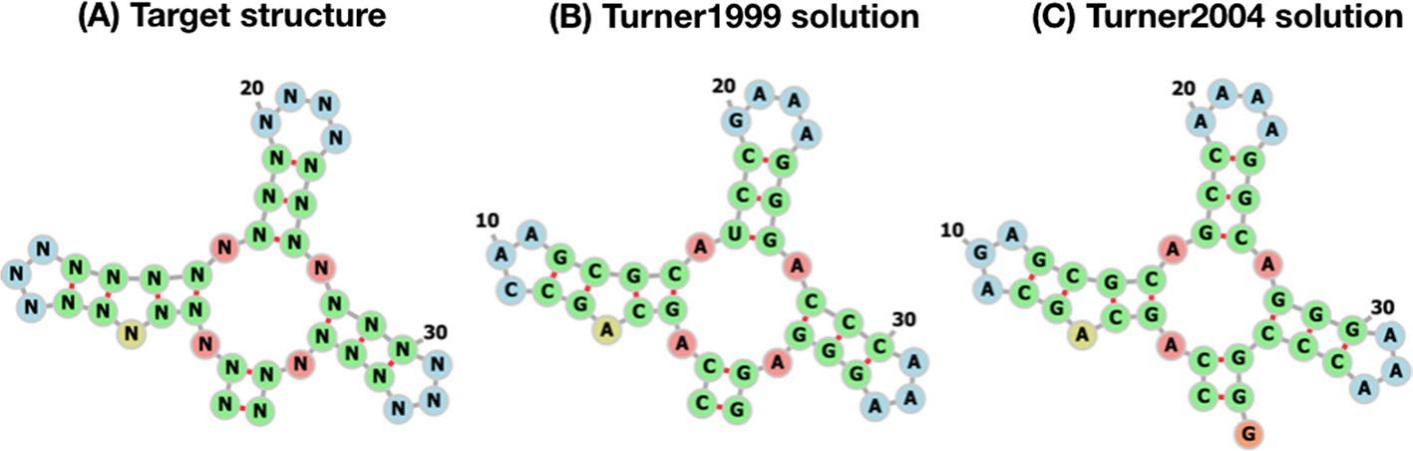
aRNAque’s performance on a TRIPOD secondary structure. **a** The tripod target structure. **b** aRNAque’s solution using the Turner1999 energy parameter sets. **c** aRNAque’s solution using the Turner2004 energy parameter sets

Finally, we performed a benchmark on a tripod target secondary structure. The tripod secondary structure was used as a third test case in the work of Ivry et al. [48], and it does not contain any pseudoknot interactions. It comprises four stems, three of which with terminal hairpins, surrounding a multibranch loop (See Fig. 10a). The tripod target structure proved very challenging, especially because of its multiloop component, which is also found in some of the unsolved Eterna100 target structures. We perform here, for both energy parameters Turner1999 and Turner2004, 100 independent designs, using a population size of 100 RNA sequences and a maximum of 5000 generations. The mutation parameters used are: $P_C=\{0.4,0.5,0.1,0,0,0\}$, $P_N=\{0.7,0.1,0.1,0.1\}$ and *c* = 1.5. When using the Turner2004 energy parameter set, none of the 100 designed RNA sequences was successful (i.e., no sequence folds exactly into the target structure after 5000 generations). Figure 10b shows one of the best solutions obtained out of 100 designed sequences when using the Turner2004, the designed sequence folds into a structure at one error base-pair distance from the target structure. In contrast, when using the Turner1999 energy parameters, aRNAque successfully designed the tripod secondary structure (See Fig. 10c). The 100 sequences designed folded exactly into the target structure with an average median number of generations of 20. When comparing both solutions to the one obtained in [48], aRNAque (with no need to change the RNA structure distance) can successfully design the multibranch loop component with one base pair error using the Turner2004 energy parameter whereas RNAinverse (with the DoPloCompare distance) failed to design the multibranch loop, and the solution is at 2 base-pair distance error.

## Discussion

In this work, we have provided an updated version of aRNAque implementing a Lévy flight mutation scheme that supports pseudoknottted RNA secondary structures. A Lévy mutation scheme offers exploration at different scales (mostly local search combined with rare big jumps). Such a scheme significantly improves the number of evaluations needed to hit the target structure while better avoiding getting trapped in local optima. The benefit of a Lévy flight over a purely local mutation search allowed us to explore RNA sequence space at all scales. Such a heavy-tailed distribution in the number of point mutations permitted the design of more diversified sequences. It reduced the number of evaluations of the evolutionary algorithm implemented in aRNAque. The main advantage of using a Lévy flight over local search is a reduction in the number of generations required to reach a target. This is because the infrequent occurrence of a high number of mutations allows a diverse set of sequences among early generations without the loss of robust local search. One consequence is a rapid increase in the population mean fitness over time and fast convergence to the target of the maximally fit sequence. To illustrate that advantage, we ran aRNAque starting from an initial population of unfolded sequences, both for a ”one point mutation” and ”Lévy mutation”.

**Fig. 11:**
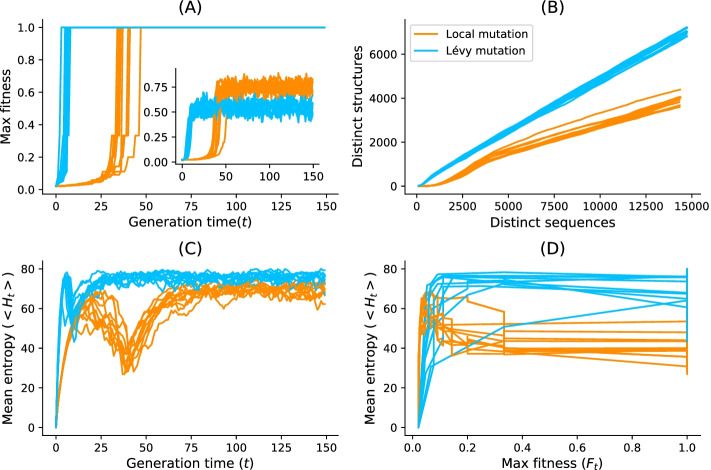
Lévy mutation vs one-point mutation. For the Eterna100 target structure *[CloudBeta] 5 Adjacent Stack Multi-Branch Loop*, ten independent runs were performed in which a minimum of 10 sequences were designed per run. **a** Max fitness and mean fitness (inset) over time. **b** Distinct sequences vs. Distinct structures over time. **c** Mean Shannon entropy of the population sequences over time for both binomial and Lévy mutation. **d** The max fitness plotted against the entropy over time

Figure 11a, b show respectively the max/mean fitness over time and the number of distinct structures discovered over time plotted against the number of distinct sequences. When using a Lévy mutation scheme, the mean fitness increases faster in the beginning but stays lower than that using local mutations. Later in the optimisation, a big jump or high mutation on the RNA sequences produces structures with fewer similarities and, as a consequence, worse fitness. In the (5–10)th generation, sequences folding into the target are already present in the Lévy flight population, but only at the 30th generation are similar sequences present in the local search population. The Lévy flight also allows exploration of both the structure and sequence spaces, providing a higher diversity of structures for any given set of sequences (Fig. 11b). Using the mean entropy of structures as an alternate measure of diversity, we see in Fig. 11c, d how a Lévy flight achieves high diversity early in implementation and maintains a higher diversity over all generations than a local search algorithm. Although the mutation parameters $P_C$ and $P_N$ influence the designed sequences’ absolute diversity, the Lévy flight always tends to achieve a higher relative diversity than the local search, all else being equal.

We argue that the improved performance of the Lévy mutation over local search in target RNA structures is due to the high base pair density of pseudoknotted structures. Given that pseudoknots present a high density of interactions, there are dramatic increases in possible incorrect folds and thus increasing the risk of becoming trapped near local optima [49]. As implied by a heavy-tailed distribution, large numbers of mutations in paired positions are necessary to explore radically different solutions.

To illustrate that Lévy Flight performance was due to base pair density, we clustered the benchmark datasets into two classes: one cluster for target structures with low base pair density (density $\le 0.5$) and a second cluster for structures with high base pair density (density $> 0.5$). Figure 10b shows the number of target sequences available in each low and high-density category. The number of targets available in each category are colored according to the percentage of pseudoknot-free targets (Eterna100-V1) vs targets with pseudoknots (Pseudobase++), showing that pseudoknots are strongly associated with high base pair densities: 71% of the pseudoknotted target structures have a high base pair density. In contrast, the Eterna100 dataset without pseudoknots has a somewhat higher representation at low base pair density. Supposing it is true that improved Lévy Flight performance is indeed tied to base pair density. In that case, it is possible that similar heavy-tailed mutation schemes could offer a scalable solution to even more complex inverse folding problems. Another measure of difficulty is the length of the target RNA secondary structure. When analysing the mean length of the pseudoknot-free targets, the high base-pair density targets are, on average, 181 nucleotides longer, and the low-density base-pair targets are 139 nucleotides (See Fig. 10c). We have 49 nucleotides for low-density targets for the pseudoknotted targets and 52 for the high-density targets. That suggests that the Lévy mutation may be a good standard for designing more challenging target structures.

Although we believe that Lévy flight-type search algorithms offer a valuable alternative to local search, we emphasise that its enhanced performance over say antaRNA is partially influenced by the specific capabilities of existing folding tools. Their limitations may account for the degradation of these tools as the pseudoknot motifs get increasingly complex (i.e. the incapacity of existing folding tools to predict some pseudoknot motifs influences the performance of both aRNAque and antaRNA). The Lévy mutation has also shown less potential in controlling the GC–content of the designed sequence when compared to antaRNA on pseudoknotted target structures. antaRNA’s parameters used in this work were tuned using pKiss; therefore, it could be possible room for improving the benchmark presented here by retuning them using IPKnot or HotKnots. Another possible limitation is the fact that most target structures were relatively easy to solve (in less than 100 generations), which possibly allowed the local search to perform better than the Lévy search in some cases. Further research on challenging target structures will improve our understanding of which conditions favour local vs. Lévy search.

## Conclusion

Our results show general and significant improvements in the design of RNA secondary structures (especially on the pseudoknotted targets) compared to the standard evolutionary algorithm mutation scheme with a mutation parameter $\approx 1/L$, where *L* is the sequence solution length. Not only does Lévy flight mutation lead to a greater diversity of RNA sequence solutions, but it also reduces the evolutionary algorithm’s number of evaluations, thus improving computing time compared to the local search. Although antaRNA average CPU time remains smaller, aRNAque’s success rate outperformed antaRNA. To further improve our program, we suggest using a more powerful computational architecture such as Massively Parallel Genetic Algorithm (MPGA). This type of architecture may allow solving more challenging target secondary structures.

### Availability and requirements

- Project name: aRNAque
- Project home page: https://github.com/strevol-mpi-mis/aRNAque
- Operating system (s): MacOS Mojave and Debian Xfce 4.12
- Programming Language: Python 3.7
- Other requirements: For pseudoknot-free target structure, please install ViennaRNA package. IPknots and Hotknots for the pseudoknotted RNA target structures.
- License: GNU GPL
- Any restrictions to use by non-academics: please contact the author at cyrillecardinale@gmail.com or csaha@aims.edu.gh.

## Supplementary Information

**Additional file 1.** Supplementary Information for aRNAque – An evolutionary algorithm for inverse pseudoknotted RNA folding inspired by Lévy flights.

## Data Availability

All the necessary data and scripts to replicate our results are available at https://github.com/strevol-mpi-mis/aRNAque.

## Abbreviations

EA: Evolutionary algorithm
RNA: Ribonucleic acid
CPU: Central processing unit
ncRNA: Non-coding RNA
lncRNA: Long non-coding RNA
sncRNA: Short non-coding RNA
tRNA: Transfert RNA
rRNA: Ribosomal RNA
MFE: Minimum free energy
MPGA: Massively parallel genetic algorithm
piRNA: PIWI-interacting RNA
snoRNA: Small nucleolar RNA
