# Supplementary material for: aRNAque: an evolutionary algorithm for inverse pseudoknotted RNA folding inspired by Lévy flights
Source: BMC Bioinformatics. 2022 Aug 13;23:335. doi: 10.1186/s12859-022-04866-w (PMC9375295; doi:10.1186/s12859-022-04866-w)
Supplement: Supplementary file 1 — Additional file 1. Supplementary Information for aRNAque – An evolutionary algorithm for inverse pseudoknotted RNA folding inspired by Lévy flights. [file 12859_2022_4866_MOESM1_ESM.pdf]

Supplementary Information for

aRNAque – An evolutionary algorithm for inverse pseudoknotted RNA folding inspired by Lévy flights

Nono S. C. Merleau, Matteo Smerlak

Corresponding author: Nono Saha Cyrille Merleau
Email: nonosaha@mis.mpg.de

1 `aRNAque's GC-content parameters`

The GC-content is controlled in `aRNAque` using the mutation parameters P_C and P_N. The following table gives the corresponding mutation parameters to the four regimes of GC-content values used for our benchmark.

Table 1: Mutation parameters used in `aRNAque` to control the GC–content values

GC–content values	P_C	P_N	`aRNAque's key`
0.25	$\{0.125, 0.125, 0.3, 0.3, 0.075, 0.075\}$	$\{0.125, 0.125, 0.375, 0.375\}$	GC25
0.25	$\{0.25, 0.25, 0.2, 0.2, 0.05, 0.05\}$	$\{0.25, 0.25, 0.25, 0.5\}$	GC50
0.75	$\{0.375, 0.375, 0.1, 0.1, 0.025, 0.025\}$	$\{0.375, 0.375, 0.125, 0.125\}$	GC75
1.0	$\{0.5, 0.5, 0.0, 0.0, 0.0, 0.0\}$	$\{0.5, 0.5, 0., 0.\}$	GC

2 Benchmark on `Eterna100` dataset

For each of the benchmarks on the `Eterna100` datasets, We ran the first benchmark using the default `aRNAque`'s parameter configuration. And then, the unsolved structures are sorted out to run a second benchmark with a maximum number of generations set at 5000. `aRNAque`'s performance presented in the paper is a combination of all the designed sequences for each realisation.

3 General EA benchmark parameters

The same hardware resources and the same computer are used for all the benchmarks listed in the following table. A supercomputer with 40-Core Intel Xeon E5-2698 v4 at 2.2 GHz and 512 GB of RAM with a Debian OS.

Table 2: Evolutionary algorithm parameter for each benchmarks.

Benchmark	Population size	Number of generations (T)	Stopping criterion	Mutation parameter	# of runs per target
PseudoBase++ (IPknot)	100	200	$t = T$ or max fitness = 0	$c = 1.5$ $P_N = \{0.7, 0.1, 0.1, .1\}$ $P_C = \{0.4, 0.5, 0.1, 0., 0., 0.\}$	20
PseudoBase++ (Hotknots)	100	200	$t = T$ or max fitness = 0	$c = 1.5$, $P_N = \{0.7, 0.1, 0.1, .1\}$ $P_C = \{0.4, 0.5, 0.1, 0., 0., 0.\}$	20
PseudoBase++ GC–content (IPknot)	100	200	$t = T$ or max fitness = 0	$c = 1.5$, $P_N = \{0.7, 0.1, 0.1, .1\}$ $P_C = \{0.4, 0.5, 0.1, 0., 0., 0.\}$	20
Tuning Parameter (Binomial,IPknot)	100	200	$t = T$ or max fitness = 0	$\mu \in [0, 0.2]$,$c = None$ $P_N = \{0.7, 0.1, 0.1, .1\}$ $P_C = \{0.4, 0.5, 0.1, 0., 0., 0.\}$	20
Tuning Parameter (Lévy, IPknot)	100	200	$t = T$ or max fitness = 0	$c \in [1, 2]$,$\mu = None$ $P_N = \{0.7, 0.1, 0.1, .1\}$ $P_C = \{0.4, 0.5, 0.1, 0., 0., 0.\}$	20
Eterna100-V1 (OP, RNAfold)	100	5000	$t = T$ or max fitness = 0	$c = 7$, $P_N = \{0.7, 0.1, 0.1, .1\}$ $P_C = \{0.4, 0.5, 0.1, 0., 0., 0.\}$	5
Eterna100-V1 (Lévy, RNAfold)	100	5000	$t = T$ or max fitness = 0	$c = 1.$, $P_N = \{0.7, 0.1, 0.1, .1\}$ $P_C = \{0.4, 0.5, 0.1, 0., 0., 0.\}$	5
Eterna100-V2 (OP, RNAfold)	100	5000	$t = T$ or max fitness = 0	$c = 7$, $P_N = \{0.7, 0.1, 0.1, .1\}$ $P_C = \{0.4, 0.5, 0.1, 0., 0., 0.\}$	5
Eterna100-V2 (Lévy, RNAfold)	100	5000	$t = T$ or max fitness = 0	$c = 1.5$, $P_N = \{0.7, 0.1, 0.1, .1\}$ $P_C = \{0.4, 0.5, 0.1, 0., 0., 0.\}$	5

4 aRNAque's evolutionary algorithm (EA)

Algorithm 1: aRNAque

/* $P' = \{S'_1 \dots S'_n\}$: the best population;
$P = \{S_1 \dots S_n\}$: the initial population of n RNA sequences;
$P_C = \{w_{AU}, w_{GU}, w_{GC}\}$: a vector containing the weights associated with each base pair;
$P_N = \{w_A, w_U, w_C, w_G\}$: a vector containing the weights associated with each nucleotide;
$\mathcal{D}$: a given probability distribution (Lévy or Binomial) with parameter p and L, where L is the length of the target RNA structure;
T: the maximum number of generations;
n: the population size ;
$f(;)$: the fitness function used. It can be the hamming, base-pair or energy distance;
σ^*: the target structure;
$\mathcal{P}$: the energy parameters used for the folding */

Input: n, T, P_N,P_C, P, $\mathcal{D}(p, L), f(;), \sigma^*, \mathcal{P}$
Output: Best population P'

$P' \leftarrow P$; // Assign the initial population to the best population
$t \leftarrow 0$; // Initialize the number of generations to 0
while $t \leq T$ & $f(S_b, \sigma^*) \neq 1$ **do**
- $\Sigma \leftarrow \{\arg\min_{\sigma \in \Gamma} E(S_i, \sigma, \mathcal{P})\}$, where $i \in \{1, 2, \dots, n\}$, Γ the structural ensemble and $E(S_i, \sigma)$ the free energy computed using the parameters $\mathcal{P}$; // Fold each sequence $S_i \in P'$ and store them in Σ
- $\kappa = \lfloor (n \times 0.1) \rfloor$; // The number of RNA sequences to copy in the next generation without mutating them.
- $F \leftarrow \{f(\sigma, \sigma^*) | \forall \sigma \in \Sigma\}$; // Evaluate the fitnesses of the folded population to the target strucre σ^* and store them in a list F
- $E_\kappa \leftarrow \{S_1 \dots S_\kappa\} \sim F$; // copy of the 10% best sequence based on their fitnesses F
- $P_S \leftarrow \{S_i\} \sim F$, where $i \in \{1, 2, \dots, n - \kappa\}$; // Randomly sample $(n - \kappa)$ RNA sequences from P' with respect to their fitnesses F
- $M \leftarrow$ mutate$(P_S, \mathcal{D}(p, L), P_C, P_N)$; // Mutated the selected sequences using the mutation algorithm presented in the main text in out paper.
- $P_b \leftarrow M \cup E_\kappa$; // Combine the mutated population and the best solutions to form the new population that will be evolved in the next generation
- $S_b \leftarrow \arg\max_{\sigma \in \Sigma} f(\sigma, \sigma^*)$;
- $t \leftarrow t + 1$; // Increment the time step (the number of generations)

The fitness function used is defined as :

$$f(\phi, \sigma*) = \frac{1}{1 + d(s^{MFE}(\phi), \sigma*)} \quad (1)$$

where $d(\cdot, \cdot)$ is the hamming distance on the structure space (structures are in dot and bracket representation).

5 Other benchmark on Eterna100-V1

The results on Eterna100-V1 presented in the paper are the best of all the benchmarks we have performed. Since our mutation scheme relies on the nucleotide distributions which implicitly control the GC–content of the designed sequences, to obtain our results, we first selected an arbitrary set of pairs $\{P_N, P_C\}$ and benchmark aRNAque on Eterna100-V1 for each of them. The success rate measures the fraction of sequences successfully folding into the target structure. Table 3 shows the different parameters we considered and the corresponded input key parameter using the call of aRNAque script. Summary of the benchmark presented in Table 4 is obtained by launching for each target structure 5 independent runs, with a population size of 100 and a maximum number of generations of 5000. The energy parameter used here was the Turner1999. The dashes in the table mean the benchmarks have not been performed for the parameters.

Table 3: Different parameters for the base pair distributions

Key	$P_N = \{p_A, p_G, p_U, p_C\}$	$P_C = \{p_{GC}, p_{CG}, p_{AU}, p_{UA}, p_{GU}, p_{UG}\}$
ALL	$P_N = \{0.25, 0.25, 0.25, 0.25\}$	$P_C = \{0.2, 0.2, 0.1, 0.1, 0.2, 0.2\}$
GC	$P_N = \{0.25, 0.25, 0.25, 0.25\}$	$P_C = \{0.5, 0.5, 0, 0, 0, 0\}$
GC_1	$P_N = \{0.25, 0.65, 0.05, 0.05\}$	$P_C = \{0.4, 0.5, 0.1, 0, 0, 0\}$
GC_2	$P_N = \{0.7, 0.1, 0.1, 0.1\}$	$P_C = \{0.4, 0.5, 0.1, 0, 0, 0\}$
GC_3	$P_N = \{0.75, 0.1, 0.1, 0.05\}$	$P_C = \{0.4, 0.5, 0.1, 0, 0, 0\}$
GC_4	$P_N = \{0.95, 0, 0.05, 0\}$	$P_C = \{0.4, 0.4, 0.2, 0, 0, 0\}$
GC_5	$P_N = \{0.7, 0.1, 0.1, 0.1\}$	$P_C = \{0.3, 0.2, 0.2, 0.1, 0.1, 0.1\}$

Table 4: Success percentage on `Eterna100` datasets for each set of mutation parameters.

Tools	BP param	Mutation param	Percentage of success	$\#(Med(gen_{Zipf}) < Med(gen_{op}))$ $\#(Med(gen_{Zipf}) > Med(gen_{op}))$
`aRNAque`	ALL	Zipf ($c = 1$)<br>One point	67%<br>81%	7(#4)<br>64(#4)
`aRNAque`	GC	Zipf ($c = 1$)<br>One point	80%<br>90%	43(#10)<br>30(#474)
`aRNAque`	GC_1	Zipf ($c = 1$)<br>One point	84%<br>90%	29(#4)<br>33(#7)
`aRNAque`	GC_2	Zipf ($c = 1$)<br>One point	89%<br>91%	61(#10)<br>19(#1920)
`aRNAque`	GC_3	Zipf ($c = 1$)<br>One point	88%<br>−−	−−<br>−−
`aRNAque`	GC_4	Zipf ($c = 1$)<br>One point	−−<br>−−	−−<br>−−
`aRNAque`	GC_5	Zipf ($c = 1$)<br>One point	82%<br>83%	44(#9)<br>30(#145)
**Total**	–	Zipf ($c = 1$)<br>One point<br>RNAinverse	**90%**<br>**92%**<br>**87%**	

6 Tools patching

To be able to perform our benchmarks, some slight modifications was made on `HotKnots` and `antaRNA`. Details about the modifications are provided in this section.

- `antaRNA`: The change was made at the line 1178 column 7, where the line args = 'HotKnots -m CC -s ' + sequence was replaced by to args = './HotKnots -m CC -s ' + sequence. The version of `antaRNA` we used is v2.0.1, and it can be found on the Github link: `https://github.com/RobertKleinkauf/antarna`.

- `HotKnots`: to run `HotKnots`, we have to move `aRNAque` to the bin directory. To avoid that, we updated the source code and recompiled a new bin that does not require to move `aRNAque` to the bin directory of `HotKnots`. We have uploaded the patched version of `HotKnots` in a third-part folder in `aRNAque`'s repository for benchmark reproduction.

  NB: The patches do not affect the folding algorithm. It consisted of avoiding the use of relative paths in `HotKnots`.
